# Implementing Community-Based Strategies for Improved Pneumonia Care in Children: Insights From a Pilot Study

**DOI:** 10.7759/cureus.58159

**Published:** 2024-04-12

**Authors:** Selvi M, Sasi Vaithilingan

**Affiliations:** 1 Department of Nursing, Vinayaka Misssions College of Nursing, Salem, IND

**Keywords:** information, community-acquired pneumonia, child health management, maternal awareness, health-seeking behaviour, community-based intervention, pneumonia prevention, child mortality, respiratory issues

## Abstract

Introduction: Respiratory ailments, encompassing a spectrum of disorders, are a leading cause of mortality and morbidity in children, with pneumonia being particularly significant, accounting for 16% of child mortality. To ensure timely engagement with healthcare services, it is imperative to instill awareness through Information, Education, and Communication (IEC) initiatives targeting mothers of children under five. The primary objective of this pilot study is to assess the feasibility of a community-based intervention on health-seeking behaviour, knowledge, and practice measures concerning the management and prevention of pneumonia in children.

Methodology: The pilot study mirrored the main study's procedures in two villages, Bhuvanahalli and Gavanahalli, each randomly assigned as either an experimental or a control group. We selected 12 mothers with children under the age of five who had community-acquired pneumonia, employing a straightforward random technique, with six mothers from each group. These mothers were interviewed using a structured questionnaire focusing on health-seeking behaviour, knowledge, and practices related to the management and prevention of pneumonia. Mothers in the experimental group received a community-based intervention, specifically an educational set focusing on health-seeking behaviour, knowledge, and practice measures concerning the management and prevention of pneumonia in children, while those in the control group continued with their routine practices. We collected post-test data from the mothers in both groups at the 2nd, 4th, and 6th months of the intervention. The data analysis was conducted using the IBM SPSS Statistics for Windows, Version 28 (Released 2021; IBM Corp., Armonk, New York) software.

The Mann-Whitney test and Kruskal-Wallis analyses indicated a notable and statistically significant shift in health-seeking behaviour, knowledge, and practices pertaining to the management and prevention of pneumonia in children as a result of the community-based educational intervention implemented in the experimental group (P<0.05).

Conclusion: Community-based intervention is crucial to preventing mortality and morbidity in children. The findings of the pilot study affirm its feasibility and lay a strong foundation for further investigation and implementation.

## Introduction

Respiratory conditions are a significant contributor to both child mortality and morbidity. Various respiratory issues affect children under the age of five, with pneumonia being the most common [1]. Pneumonia is characterized by the consolidation and exudation of lung tissue. Multiple factors contribute to the onset of childhood pneumonia, including seasonal variations, inadequate ventilation, substandard living conditions, cross-infections, low immunity, and insufficient child care [1,2]. The prevention of community-acquired pneumonia is heavily reliant on the role of caregivers, particularly mothers [3,4].

Pneumonia in children is a preventable condition and can be addressed through immunization, adequate balanced nutrition, and maintaining a hygienic environment [5]. The World Health Organization's global prevention of pneumonia and diarrhoea in children emphasizes the need to enhance access to healthcare, improve nutrition, promote exclusive breastfeeding, and upgrade living conditions to reduce the burden of childhood pneumonia. It also suggests that counselling interventions at the community doorstep and in healthcare facilities such as health centres and hospitals could be beneficial in reducing the incidence of community-acquired pneumonia (CAP) [6].

Pneumonia and diarrhoea are recognized as significant contributors to child mortality, accounting for 29% of deaths in children under five [6,7]. Globally, almost 2 million children under five die each year due to pneumonia [6,7]. The challenge of regional inequities is pronounced in low- and middle-income countries, where 81% of deaths due to severe pneumonia occur outside hospitals, mainly due to delayed access to healthcare services [8,9]. In addition to mortality, pneumonia contributes to morbidity in children due to the long-term chronicity of lung dysfunction. Severe or recurrent pneumonia can result in chronic impairment of lung tissue [9]. Lower respiratory tract infections are typically uncomplicated, yet they can lead to serious complications, including, but not limited to, congestive heart failure, respiratory failure or arrest, and sepsis, resulting in collapse and lung abscesses. Children under five are a group at a high risk for complications [8,9].

To effectively address pneumonia and diarrhoea-related mortality in India, it is essential to understand their causes. Recent study analysis combined time series evaluations of seasonal patterns, climatic regions, and clinical symptoms based on 243,000 verbal autopsies from the nationally representative Million Death Study. In this study, the authors found that pneumonia mortality among children aged 1 month to 14 years was highest in January, with a rate ratio (RR) of 1.66 (99% confidence interval (CI) 1.51-1.82) compared to the lowest rate in April. The increased RRs observed in infants aged 1-11 months suggest a possible link to respiratory syncytial virus (RSV) as a cause. Notably, India's humid subtropical region exhibited a distinct pattern of summer pneumonia mortality. Diarrhoea mortality peaked in July (RR 1.66, 1.48-1.85) and January (RR 1.37, 1.23-1.48), while deaths associated with fever and bloody diarrhoea, indicative of an enteroinvasive bacterial cause, showed minimal seasonal variation. By integrating mortality data for children aged 1-59 months with prevalence surveys, the authors of this study estimated that in 2015, there were approximately 40,600 pneumonia deaths due to *Streptococcus pneumoniae*, 20,700 due to RSV, 12,600 due to influenza, and 7,200 due to *Haemophilus influenzae* type B, as well as 24,700 diarrhoeal deaths attributable to rotavirus [10]. Thorough mortality studies are crucial for identifying the causes and guiding the introduction of vaccines.

In India, acute respiratory infections pose a significant public health challenge, particularly for children aged 0-5 years, accounting for 15%-30% of deaths in this age group [11]. Many of these deaths are preventable. A study aimed to evaluate the awareness and understanding of childhood pneumonia among mothers with children under five years of age. The cross-sectional study was conducted with 460 mothers of children under five, using an interviewer-administered structured questionnaire and employing a three-stage cluster sampling method. The questionnaire was divided into three sections: sociodemographic profile, level of knowledge, and level of perception. The study revealed that most mothers were secondary school graduates (32.6%), with 93.7% being homemakers. Approximately 41.3% of mothers had a fair level of knowledge, and 41.5% had a fair perception of pneumonia. There was a significant correlation between the mothers' age and education level and their knowledge and perception of the disease. Additionally, a significant association was found between the level of knowledge and perception of childhood pneumonia among these mothers [11]. Overall, the mothers exhibited a fair understanding and perception of childhood pneumonia. However, there is a need to address the lack of awareness regarding simple signs, symptoms, and risk factors associated with pneumonia.

The perception and healthcare-seeking behaviours of mothers for their under-five children in rural Bangladesh revealed that a majority of rural mothers lacked sufficient and accurate knowledge and perceptions concerning childhood pneumonia. Alarmingly, many of these mothers did not seek appropriate care even when their children were suffering from severe or very severe pneumonia. It recommended the implementation of health education initiatives, either at the household or community level, or through mass education campaigns, to disseminate information to warrant immediate treatment at healthcare facilities [12].

Despite government initiatives to provide healthcare facilities for children, the reachability of these services is crucial. Lack of awareness of the available facilities, lack of knowledge on identification, management, and prevention of pneumonia, and lack of consistent practice of household and environmental hygienic measures by mothers pose a threat to CAP in children under five.

Based on the identified gaps in existing knowledge and practice regarding the management and prevention of CAP in children under five, this study aims to evaluate the impact of a community-based intervention. This intervention is designed to improve the health-seeking behaviour, knowledge, and practice of mothers with under-five children who are diagnosed with CAP. The community-based intervention encompasses a series of educational sessions, awareness campaigns, and hands-on training focused on effective pneumonia management, prevention strategies, and the importance of timely healthcare engagement.

As a preliminary step, this study was conducted as a pilot version on a small scale to assess the feasibility and potential effectiveness of the intervention. The pilot study involved a select group of mothers in a controlled setting, allowing for a detailed evaluation of the intervention's impact on their health-seeking behaviour, knowledge, and practices related to pneumonia management and prevention. The results of this pilot study will be used to refine the intervention and inform the design of a larger-scale study to further investigate its effectiveness in a broader population.

## Materials and methods

Approval was obtained from the Government Medical College, Hassan, for our study (reference number IEC/HIMS/RR 290/28-06-2022). We chose mothers from Bhuvanahalli and Gavanahalli villages in the Hassan district, Karnataka, India, for our small pilot study. We randomly picked one group for the intervention. We selected mothers with children under five who had pneumonia from each village using a simple random method. In our study, the "simple random technique" referred to the process of randomly selecting mothers from the population of interest (mothers with children under five years old affected by CAP) to participate in the study. This was achieved using a random number generator to ensure that each mother had an equal chance of being selected for either the experimental or control group. The random assignment was performed to minimize selection bias and ensure that the groups were comparable at baseline. The study lasted six months, from July to December 2022. We collected data from the intervention group in the 2nd, 4th, and 6th months. The main study will have 60 mothers in each group, but for this pilot, we chose six mothers from each group as a starting point. We included mothers of children under five diagnosed with pneumonia who could speak and understand Kannada. We did not include mothers with children who had severe pneumonia or other lung problems to keep our study accurate.

We conducted interviews with the mothers using a set of questions that we developed. The development of the questionnaire was a meticulous process that involved a comprehensive review of the literature and existing validated instruments related to pneumonia knowledge, health-seeking behaviour, and preventive practices [9-12]. The questions were carefully framed to ensure relevance, clarity, and appropriateness for the target population of mothers with children under five years old. A panel of experts in paediatrics, public health, and epidemiology reviewed the questionnaire to determine its validity and reliability. Their feedback was instrumental in refining the questionnaire. Furthermore, a pretest was conducted with a small sample of mothers from a similar demographic but not included in the main study. This pretest allowed us to assess the understanding of the questions and the overall flow of the questionnaire and to make necessary adjustments based on the feedback received. So, the final questionnaire was checked and approved for use in the main study, which looked at how the community-based intervention changed mothers' health-seeking behaviour, knowledge, and practices about how to treat and prevent pneumonia in children younger than five. We wrote the questions in English, translated them into Kannada, and then translated them back to English to ensure they were clear. We checked the reliability of our questions about healthcare-seeking behaviour and practices using a method that compares different raters. The reliability scores were 0.89 for the healthcare-seeking behaviour questions and 0.92 for the practice questions, showing they were reliable. We also used the split-half method for the knowledge questions and got a reliability score of 0.87, which is good. Experts helped us make sure the questions were valid.

The set of questions had four parts: Part A asked for basic information on the mother and child, their social and economic status, and their living conditions. Part B asked how they seek healthcare; Part C asked about their knowledge of how to manage and prevent pneumonia in children; and Part D asked how they practice handwashing and keeping their environment clean.

After we collected data for the first time, we gave the mothers flashcards at their homes to help them learn more about managing and preventing pneumonia, recognizing its danger signs, and keeping clean. We showed them how to wash their hands and inhale steam. We asked them to follow these steps for a week, and then we checked on them after 2, 4, and 6 months from the first data collection. We used the IBM SPSS Statistics for Windows, Version 28 (Released 2021; IBM Corp., Armonk, New York) software to analyze the data. We talked about data that can be counted using frequencies and percentages and data that can be measured using means, medians, and ranks. We compared data within each group using the Kruskal-Wallis test and between groups using the Mann-Whitney U test.

## Results

As the sample size was small, non-parametric tests were used for analysis. In Table 1, Fisher's exact test for the demographic characteristics of mothers of under-five children showed no significant differences between the experimental and control groups for all included characteristics.

**Table 1:** Distribution of sociodemographic variables between the experimental and control groups P>0.05: non-significant

Sociodemographic Variables	Experimental Group	Control Group	Fisher
	N (%)	N (%)	Exact Value
1. Age of the mother			
20–25 years	2 (33.33)	4 (66.67)	
26–30 years	3 (50)	2 (33.33)	
31–35 years	0	0	0.5
Above 35 years	1 (16.7)	0	
2. Religion			
Hindu	5 (83.3)	5 (83.3)	1
Christian	0	0	
Muslim	1 (16.7)	1 (16.7)	
3. Type of family			
Nuclear	3 (50)	3 (50)	1
Joint	3 (50)	3 (50)	
4. Monthly income of the family	1 (16.7)	1 (16.7)	1
Rs. 47,348 and above			
Rs. 23,674–47,347	1 (16.7)	1 (16.7)	
Rs. 17,756–23,673	0	0	
Rs. 11,837–17,755	2 (33.3)	2 (33.3)	
Rs. 7102–11,836	1 (16.7)	1 (16.7)	
Rs. 2391–7101	1 (16.7)	1 (16.7)	
5. Educational status of the mother	1 (16.7)	1 (16.7)	1
Illiterate			
Primary education	1 (16.7)	1 (16.7)	
High school education	2 (33.3)	2 (33.3)	
Higher secondary education	1 (16.7)	1 (16.7)	
Diploma and above	1 (16.7)	1 (16.7)	
6. Occupation of the mother	2 (33.3)	2 (33.3)	1
Semi-skilled worker			
Unskilled worker	2 (33.3)	2 (33.3)	
Unemployed	2 (33.3)	2 (33.3)	
7. Number of children			1
One	2 (33.3)	2 (33.3)	
Two	4 (66.67)	4 (66.67)	
8. Experience of respiratory infections			1
Yes	2 (33.3)	2 (33.3)	
No	4 (66.67)	4 (66.67)	
9. Source of information			
Newspaper	1 (16.7)	1 (16.7)	
Mass media	0	0	1
Health professional	4 (66.67)	4 (66.67)	
Family members	0	0	
Others	1 (16.7)	1 (16.7)	
10. Immunization status as on age (vaccination status of the child based on their age-appropriate immunization schedule)			
Yes	5 (83.3)	5 (83.3)	1
No	1 (16.7)	1 (16.7)	
11. Type of house			
Permanent house (pucca)	3 (50)	5 (83.3)	
Semi-permanent (semi-pucca)	2 (33.3)	0	1
Temporary house (katcha)	1 (16.7)	1 (16.7)	
12. Presence of a separate kitchen			
Yes	5 (83.3)	5 (83.3)	1
No	1 (16.7)	1 (16.7)	
13. Information about environmental health issues			
Local newspaper	1 (16.7)	1 (16.7)	
Social media	1 (16.7)	1 (16.7)	1
Healthcare provider	4 (66.67)	4 (66.67)	
14. Using wood stoves in our home			
Yes	3 (50)	3 (50)	1
No	3 (50)	3 (50)	
15. Presence of an exhaust fan in the kitchen			
Yes	5 (83.3)	5 (83.3)	1
No	1 (16.7)	1 (16.7)	

Similarly, Table 2 shows a significant difference (P<0.05) in the post-test mean ranks between the experimental and control groups when looking at health-seeking behaviour using the Mann-Whitney U test.

**Table 2:** Comparison of health-seeking behaviour between the experimental and control groups in the post-tests Mann-Whitney U test for non-parametric data EG: experimental group; CG: control group; NS: non-significant; S: significant

Sociodemographic Variables	Median Rank-EG	Median Rank-CG	U-Value	P-Value	Significance	
						1. Recently sought healthcare facility						
Yes	5.5	3.5	0.4	0.3	NS	
No	3.5	5.5	0.4	0.3	NS	
2. Decision about child treatment						
Previous experience	2.5	6.5	0	0.01	S	
Parents	6	3	2	0.04	S	
Grandparents	2.5	6.5	0	0.01	S	
Health worker	6	3	2	0.04	S	
3. Type of preferences						
Allopathic medicine	5.12	3.88	5.5	0.43	NS	
Alternative medicine	4.5	4.5	8	1	NS	
Traditional healers	4.5	4.5	8	1	NS	
4. Type of utilization of health service						
Government hospitals	2.5	6.5	0	0.01	S	
Government clinics	4.5	4.5	8	1	NS	
Private hospitals	2.5	6.5	0	0.01	S	
Private clinics	6.5	2.5	0	0.01	S	
5. Stage of illness on admission						
Breathing difficulty	5	4	6	0.68	NS	
Episodes of cough	5	4	6	0.68	NS	
Chest in-drawing	4	5	6	0.68	NS	
Refusal of feeds	4.5	4.5	8	1	NS	
Fever	2.5	6.5	0	0.08	NS	
Vomiting	4.5	4.5	8	1	NS	
6. Onset of symptoms and seeking healthcare						
After 1–2 days	6	3	2	0.04	S	
After 3–4 days	3	6	2	0.04	S	
7. Reason for delay in seeking healthcare						
Cost of consultation and treatment	6	3	2	0.04	S	
Both parents are working	6.5	2.5	0	0.01	S	
Long waiting time	6.5	2.5	0	0.01	S	
Appointment issues	2.5	6.5	0	0.01	S	
No competent physician nearby	2.5	6.5	0	0.01	S	
Others, specify	2.5	6.5	0	0.01	S	

Notable changes were observed in the health-seeking behaviour of mothers in the experimental group concerning decision-making about child treatment, type of health service utilization, and reasons for the delay in seeking healthcare. This suggests that the intervention effectively improved the health-seeking behaviour of mothers of under-five children with pneumonia in the experimental group.

Furthermore, after the intervention, parents and health workers became the primary decision-makers in the experimental group, while grandparents remained the primary decision-makers in the control group (P<0.05). Only after their child had pneumonia did mothers in the control group seek medical attention. In contrast, mothers in the experimental group utilized private clinics for health services. Mothers in the control group used both public and private hospitals (P<0.05). Mothers in the experimental group sought healthcare for their children within two days of illness, compared to the control group. The cost of consultation and treatment, both parents working, and long waiting times were the reasons for the delay in seeking healthcare among mothers in the experimental group. Appointment issues and the lack of competent physicians were reasons for the delay in the control group.

The pre-test knowledge of mothers was assessed using mean ranks, which were 5.83 for the experimental group and 7.17 for the control group. The Mann-Whitney test (U=14, P>0.05) revealed no significant difference in knowledge before the intervention between the groups. Similarly, the pre-test mean rank for practice was 5.17 for the experimental group and 7.83 for the control group, with no significant difference (U=10, P>0.05) between the groups. Table 3 compares the mean rank of knowledge at different points of assessment using the Kruskal-Wallis test, revealing a significant difference (19.5, P<0.05) in the knowledge of mothers in the experimental group on the management and prevention of pneumonia after the intervention.

**Table 3:** Effectiveness of intervention on knowledge regarding management and prevention of pneumonia in children P<0.001 represents the statistical significance of the differences observed either within a group over time (pre-test to post-test) or between the experimental and control groups at each time point. Mann-Whitney U test for non-parametric data and the Kruskal-Wallis test for comparisons within groups over time. Post-test data were collected at the 2nd, 4th, and 6th months of the intervention.

Assessments	N	Experimental Group	Control Group	P-Value
		Mean Rank	Mean Rank	
Pre-test	6	3.5	8.92	<0.001
Post-test 1	6	10.5	11.92	<0.001
Post-test 2	6	20.5	15.42	<0.001
Post-test 3	6	15.5	13.75	<0.001

In contrast, mothers in the control group showed no significant improvement (2.89, P>0.05) in their knowledge. Table 4 depicts a statistically significant difference (20.18, P<0.05) in the practice of mothers regarding the management and prevention of pneumonia after the intervention at different time points of post-assessment in the experimental group.

**Table 4:** Effectiveness of intervention on practice regarding management and prevention of pneumonia in children P<0.001 represents the statistical significance of the differences observed within a group over time (pre-test to post-tests). ^#^Denotes P<0.001, indicating a significant difference between the experimental and control groups in the pre-test and post-test 3. ^$^Denotes, P>0.05, indicating no significant difference between the experimental and control groups in post-test 1 and post-test 2. Mann-Whitney U test for non-parametric data and the Kruskal-Wallis test for comparisons within groups over time.

Data Points	N	Experimental Group	Control Group	P-Value
		Mean Rank	Mean Rank	
Pre-test	6	3.5	10.25	<0.001^#^
Post-test 1	6	11.33	11.92	>0.05^$^
Post-test 2	6	13.67	12.67	>0.05^$^
Post-test 3	6	21.5	15.17	<0.001^#^

However, the practice of mothers in the control group did not show a significant difference (0.677, P>0.05) in the post-assessments. Further analysis revealed no significant association between pre-test knowledge and practice with selected sociodemographic variables.

## Discussion

The baseline data provided offer a comprehensive understanding of the sociodemographic variables among mothers of children with pneumonia in both the experimental and control groups. This information is crucial in assessing the initial similarities and differences between the groups, ensuring a balanced starting point for any intervention or study.

The sociodemographic variables for both the experimental and control groups are similar. The Fisher exact value obtained indicates no significant difference in the distribution, suggesting that both groups are homogeneous.

In both the experimental and control groups, approximately 50% of the participants were aged 26-30 years. The majority were Hindus, with 50% coming from nuclear families and 50% from joint families. Approximately one-third had a monthly income of Rs 11,837-17,755, and their educational status fell into the high school category. The mothers' occupational status was equally categorized into skilled, semi-skilled, and unskilled. Most had two children, and the majority received information from health professionals. Nearly all the children were immunized. The mothers resided in both permanent (pucca) and temporary (katcha) houses, with the majority receiving health-related information from healthcare providers. Most had an exhaust fan in their kitchen.

The pre-test values show that both the experimental and control groups had similar levels of knowledge and practice. The Mann-Whitney U test analysis indicates no significant difference in the mean rank between the groups (P>0.05).

A study assessed the knowledge regarding pneumonia among parents of under-five children in selected hospitals in Pune City. The study revealed that pneumonia kills a child every 39 seconds globally, making it more lethal than any other infectious disease, with approximately 80,000 deaths per year and 2,200 per day. Over 153,000 newborns are included. In comparison, in 2018, approximately 437,000 children under five died due to diarrhoea and 272,000 due to malaria [1]. The study used a non-probability purposive sampling technique with 100 samples. The demographic characteristics showed that the majority of participants were aged 25-30, with 59% of mothers present with their child, 54% belonging to nuclear families, 41% of mothers with secondary education, 25% of mothers self-employed, 36% of fathers with secondary education, and 53% of fathers self-employed. The study concluded that parents of under-five children have adequate knowledge regarding pneumonia [1].

Overall, the baseline data demonstrate a striking similarity in sociodemographic characteristics, pre-test values, and healthcare-related variables between the experimental and control groups. This homogeneity lays a robust foundation for subsequent comparative analyses, ensuring that any observed changes or outcomes can be more confidently attributed to the intervention or control measures rather than initial group disparities.

The effectiveness of community-based intervention on pneumonia was evaluated. The knowledge of mothers in the experimental group showed a significant increase in the post-test, with mean ranks of 3.5, 10.5, 20.5, and 15.5 in the pre-test, post-test 1, post-test 2, and post-test 3, respectively. The Kruskal-Wallis value (19.5, P<0.05) indicates a significant increase in knowledge related to pneumonia in the experimental group, suggesting that community-based intervention is very effective.

In contrast, the knowledge of mothers in the control group showed no significant change, with mean ranks of 8.92, 11.92, 15.42, and 13.75 in the pre-test, post-test 1, post-test 2, and post-test 3, respectively. The Kruskal-Wallis value (2.89, P>0.05) indicates no significant change in knowledge related to pneumonia.

The practice of mothers in the experimental group showed a significant increase, with mean ranks of 3.5, 11.33, 13.67, and 21.5 in the pre-test, post-test 1, post-test 2, and post-test 3, respectively. The Kruskal-Wallis value (20.18, P<0.05) indicates a significant increase in pneumonia-related practice, suggesting that community-based intervention is very effective.

However, the practice of mothers in the control group showed no significant change, with mean ranks of 10.25, 11.92, 12.67, and 15.17 in the pre-test, post-test 1, post-test 2, and post-test 3, respectively. The Kruskal-Wallis value (0.677, P>0.05) indicates no significant change in pneumonia-related practice.

The results supported by a factorial design conducted in Lucknow, India, showed that a village-based intervention on pneumonia was more effective (79.3%, P<0.01) than a facility-based intervention (68.9%, P<0.005). The study concluded that a structured awareness program on pneumonia was valid and informative [13]. These findings suggest the underpinning role of various factors, such as procalcitonin and C-reactive protein in CAP [14,15].

Sahoo et al. (2021) aimed to assess and enhance the knowledge of mothers with children under five years old regarding the prevention of pneumonia. A total of 20 samples were selected from Kalinga Institute of Medical Sciences (KIMS) and Pradyumna Bal Memorial Hospital (PBMH) in Bhubaneswar, Odisha, using purposive sampling. The participants, aged 18-37 years with children below the age of 5, were given a closed-ended questionnaire to assess their knowledge about pneumonia. Additionally, an information booklet on pneumonia prevention was provided [16]. The results showed that the mothers' knowledge of pneumonia was very poor in the pre-test but significantly improved in the post-test. Most of the mothers were aged 28-35 years, with most having a graduation level of education. Most of the participants were Hindu, housewives, and belonged to middle-class families living in rural areas, with a monthly income ranging from 1001 to 5000. In the pre-test, the overall level of knowledge about pneumonia among the mothers was 40%, which increased to 70% in the post-test [16]. This suggests that the intervention was successful in improving the understanding of pneumonia prevention among mothers with children under five years old. Our results are consistent with prior research on the benefits of early interventions in CAP [17,18].

They utilized a closed-ended questionnaire to evaluate pneumonia-related knowledge pre- and post-intervention. Information on pneumonia prevention was provided through an information booklet. The pre-test revealed poor knowledge among the subjects, but knowledge significantly improved post-intervention, showing good scores.

In this pilot version of the study, higher statistical analysis could not be used due to the small sample size. However, the pilot study demonstrated that the implementation of interventions at the community level was feasible and practicable.

## Conclusions

The study underscores the effectiveness of community-based interventions in enhancing the knowledge and practices related to pneumonia prevention among mothers with children under the age of five. The initial data demonstrated that the experimental and control groups were comparable in terms of sociodemographic characteristics, pre-test scores, and healthcare-related variables, allowing for a robust comparison between the groups. The notable improvement in knowledge and practice in the experimental group highlights the importance of targeted educational initiatives. Furthermore, the comparison between facility-based and village-based interventions showed that the latter was more effective, underscoring the significance of local, community-driven approaches. The pilot study in Bhubaneswar further supports the notion that structured awareness programmes can significantly enhance pneumonia-prevention knowledge among mothers, contributing to better child-rearing practices and overall health outcomes. These findings underscore the critical role of community-based interventions and awareness programmes in addressing the global challenge of pneumonia, particularly in resource-limited settings.

## Appendices

Instructions:

Please give your response to all the items given below by placing a tick mark against the appropriate answer.

Name of the village:

House No.:

Street No.:

Name of the mother:

**Table 5:** Practice assessment scale

Hand Washing							
Sl. No.	Areas	Not yet all		A little	Moderate	Very much	Extreme
	Wash hands before and after eating, using the toilet, playing with mud, and coming into contact with an infected person.						
	Wet hands with tap water and use mild soap.						
	Rub hands together for about 20 seconds and scrub between fingers on the tops and wrists.						
	Rinse hands under tap water.						
	Dry hands with a clean towel.						
Steam inhalation							
	Wash hands and heat up the water to boiling. Steam 10–15 minutes for each session. Carefully pour the hot water into the bowl.						
	Be extremely careful to avoid making direct contact with the hot water. Inhale slowly and deeply through the nose for at least 5–15 minutes.						
	Wipe the face and neck properly using a hand towel.						
	Have the child remain in the Fowler’s position or at rest for 1–2 hours after inhalation.						
	Observe for the present signs and symptoms after steam inhalation.						
Extreme – 4; Very much – 3; Moderate – 2; A little – 1; Not yet all – 0							

**Table 6:** Structured questionnaire

Part	Section	Question	Options
A	Baseline Data Of The Mother	Age Of The Mother	20-25 Years, 26-30 Years, 31-35 Years, Above 35 Years
A	Baseline Data Of The Mother	Religion	Hindu, Christian, Muslim, Others
A	Baseline Data Of The Mother	Type Of Family	Nuclear, Joint, Single-Parent Family, Extended Family
A	Baseline Data Of The Mother	Monthly Income Of The Family	Rs. 47,348 And Above, Rs. 23,674–47,347, Rs. 17,756–23,673, Rs. 11,837–17755, Rs. 7102 - 11,836, Rs. 2391–7101, Less Than Rs. 2390
A	Baseline Data Of The Mother	Educational Status Of The Mother	Illiterate, Primary Education, High School Education, Higher Secondary Education, Diploma And Above
A	Baseline Data Of The Mother	Diet	Vegetarian, Non-Vegetarian
A	Baseline Data Of The Mother	Occupation Of The Mother	Professional, Semi-Professional, Clinical/Shop/Farm, Skilled Worker, Semi-Skilled Worker, Unskilled Worker, Unemployed
A	Baseline Data Of The Mother	No. Of The Children In The Family	One, Two, Three And Above
A	Baseline Data Of The Mother	Have You Experienced With Respiratory Tract Infections	Yes, No
A	Baseline Data Of The Mother	Source Of Health Information Regarding Pneumonia	Newspaper, Mass Media, Health Professional, Family Members, Others
A	Baseline Data Of The Mother	Whether The Child Was Immunized Appropriately For The Age	Yes, No
A	Baseline Data Of The Mother	Did The Child Get Immunized With Pneumococcal Vaccine	Yes, No
A	Baseline Data Of The Mother	What Is The Type Of The House You Live In?	Pucca, Semi-Pucca, Katcha
A	Baseline Data Of The Mother	Are You Satisfied With Your Current Living Arrangement?	Yes, No
A	Baseline Data Of The Mother	Do You Have Separate Space For Kitchen In Your House?	Yes, No
A	Baseline Data Of The Mother	Where Do You Get Information About Local Environmental Health Issue?	Local/ Regional Television, Local Newspaper, Social Media, Healthcare Provider, Others
A	Baseline Data Of The Mother	Do You Have A Wood Stove In Your Home?	Yes, No
A	Baseline Data Of The Mother	Do You Have Exhaust Fan In Your Kitchen?	Yes, No
B	Health-Seeking Behaviour	In The Past Two Weeks, Did You Seek For Healthcare Facility To Manage Your Child’s Respiratory Problem	Yes, No
B	Health-Seeking Behaviour	Who Decides The Treatment For Your Child’s Illness	Previous Experience, Parents, Grandparents, Health Workers, Others
B	Health-Seeking Behaviour	Which Type Of Care You Prefer To Give For Treating Your Child’s Respiratory Problem	Allopathic Medicine, Alternative Medicine, Traditional Healers, Others
B	Health-Seeking Behaviour	Which Type Of Healthcare Facility Preferred?	Government Hospitals, Government Clinics, Private Hospitals, Private Clinic, Others
B	Health-Seeking Behaviour	At What Stage Of Illness You Take The Child To A Healthcare Facility	Breathing Difficulty, Episodes Of Cough, Chest In-Drawing, Refusal Of Feeds, Fever, Vomiting, Others
B	Health-Seeking Behaviour	Within How Many Hours/Days Of Observation Of The Symptom Of The Child Was They Taken To A Healthcare Facility	On The Same Day, After 1 To 2 Days, After 3 To 4 Days, More Than 4 Days
B	Health-Seeking Behaviour	What Was The Reason For Delay In Seeking Healthcare	Distance Of Healthcare Facility, Inadequate Transport Facilities, Cost Of Consultation And Treatment, Both Parents Are Working, Long Waiting Time, Appointment Issues, No Competent Physician Nearby, Others
C	General Concept Of Pneumonia	Following Are The Main Functions Of The Nose Except	Smell, Filtering The Inhaled Air, Humidifies The Inhaled Air, Produces Sound
C	General Concept Of Pneumonia	One Of The Following Is Essential For Life	Carbon Dioxide, Nitrogen, Oxygen, Hydrogen Peroxide
C	General Concept Of Pneumonia	Which Gas Is Coming Out From Our Body Respiration?	Carbon Dioxide, Nitrogen, Oxygen, Helium
C	General Concept Of Pneumonia	What Is Pneumonia?	A Lung Infection, A Severe Chest Cold, Infection Of The Stomach, Nose Infection
C	General Concept Of Pneumonia	One Of The Following Is The Cause Of Pneumonia	Bacteria, Viruses, Fungi, And Protozoa, Viruses Only, Fungi Only, Protozoa Only
C	General Concept Of Pneumonia	One Of The Following Group Is Most At Risk For Developing Pneumonia	Infants/Young Children (0–12 Years), Adolescents (13–19 Years), Adults (20–59), Elderly (60 And Above)
C	General Concept Of Pneumonia	How Respiratory Infection Is Transmitted To Other Children?	Common Colds, From Infected Person, Air Pollution, From Dust
C	General Concept Of Pneumonia	What Is The Main Cause Of Common Colds?	More Intake Of Water, Infections, Fasting, Malnutrition
C	General Concept Of Pneumonia	What Are The Symptoms Of Pneumonia?	Cough, Fever, And Chills; Rash, Painful Joints, And Itching Skin; Peeling Skin, Stomach Pain, And Vomiting
C	General Concept Of Pneumonia	What Is The Main Complication Of Common Colds?	Infections Of The Ears, Infections Of The Eye, Swelling Of The Feet, More Urination
C	General Concept Of Pneumonia	Following Are The Signs And Symptoms Of Pneumonia In Children Except	Abnormal Sound In The Lungs, Fever, Abnormal Breathing, Cough
C	General Concept Of Pneumonia	What Are The Signs And Symptoms Of Common Colds?	Passing Urine Many Times, Headache And Constipation, Diarrhoea And Abdominal Pain, Runny Nose, Cough, And Fever
C	General Concept Of Pneumonia	At Home The Children Are Diagnosed As Pneumonia Based On	Signs And Symptoms, Lab Tests, Child's Behaviour, Family Members
C	General Concept Of Pneumonia	Which Is The Following Complication Of Pneumonia?	Collection Of The Air In The Abnormal Cavity, Discharge From The Air, Swelling Of The Feet, Collection Of The Air In The Chest Cavity
C	Management And Prevention	What Are The Measures To Be Followed To Get Relief From Common Cold?	Fasting, Self Medication, Regular Exercise, Milk With Turmeric Powder, Steam Inhalation, And Eating Tulasi Leaves, Milk With Eggs, Hot Fermentation, And No Rice, Sugar Diet, Less Intake Of Water, Salty Food
C	Management And Prevention	What Are The Measures To Be Followed To Prevent Pneumonia?	Hand Washing, Breathing Exercise, And Hygienic Measures, Drinking Milk, Eating More Rice, Eating Egg Daily
C	Management And Prevention	How To Reduce The Recurrence Of Pneumonia Among Children's?	Balanced Diet, Hand Wash, And Personal Hygiene, Playing With Other Children, Studying Well, Eating With Family Members
C	Management And Prevention	The Physical Activity That Improve Breathing Except	Hot Air Balloon, Tongue Tube, Dragon Fire Breaths, Tumble Dryer, Running
C	Management And Prevention	What Type Of Diet Should Be Given For A Child With Respiratory Tract Infections?	Fluid Diet, Salt-Restricted Diet, Sugar-Free Diet, Well-Balanced Diet
C	Management And Prevention	Which Vitamin Gives Immunity To The Children?	Vitamin A, Vitamin B, Vitamin D, Vitamin C
C	Management And Prevention	Well-Balanced Diet Means	Taking Diet Regularly, Increasing More Food In The Diet, Contains All Nutrients, Small Quantity Of Food
C	Management And Prevention	Breathing Exercise Can Help To	Clear The Fluid From Lungs, Strengthen Diaphragm, Inhale More Air, Strengthen The Body
C	Management And Prevention	The Primary Preventive Strategy For Pneumonia Is	Antibiotic Therapy, Hand Wash, Healthy Lifestyle, Immunization With Pneumococcal And Influenza Vaccines
C	Management And Prevention	What Is The Treatment For Pneumonia?	Antibiotic Medications, Oxygen Therapy, Pain Killer Medication, Both A And B
D	Observational Checklist For Practice	Brushed The Teeth	Yes, No
D	Observational Checklist For Practice	Bathed	Yes, No
D	Observational Checklist For Practice	Wearing Neat And Clean Dress	Yes, No
D	Observational Checklist For Practice	Finger Nails Trimmed	Yes, No
D	Observational Checklist For Practice	Washing Clothes Everyday	Yes, No
D	Observational Checklist For Practice	Safe Water Supply At Home	Yes, No
D	Observational Checklist For Practice	Facilities For Disposable Of Waste	Yes, No
D	Observational Checklist For Practice	Adequate Lighting & Adequate Ventilation	Yes, No
D	Observational Checklist For Practice	Evidence Of Disease Vectors In Premises	Yes, No
D	Observational Checklist For Practice	Uncooked Food Items Placed On Bare Floor	Yes, No
D	Practice Assessment Scale	Wash Hand Before And After Food Toilet And Play With Mud And Contact With Infected Person	Not Yet All, A Little, Moderate, Very Much, Extreme
D	Practice Assessment Scale	Wet Hands In The Tap Water & Use Mild Soap	Not Yet All, A Little, Moderate, Very Much, Extreme
D	Practice Assessment Scale	Rub Hands Together For About 20 Seconds & Scrubs Between Fingers On The Tops And Writs	Not Yet All, A Little, Moderate, Very Much, Extreme
D	Practice Assessment Scale	Rinse Hands Under Tap Water	Not Yet All, A Little, Moderate, Very Much, Extreme
D	Practice Assessment Scale	Dry Hands With Clean Towel	Not Yet All, A Little, Moderate, Very Much, Extreme
D	Practice Assessment Scale	Wash Hands & Heat Up The Water To Boiling. Steam 10–15 Minutes For Each Session	Not Yet All, A Little, Moderate, Very Much, Extreme
D	Practice Assessment Scale	Be Extremely Careful To Avoid Making Direct Contact With The Water. Inhale Slowly And Deeply Through The Nose For At Least 5–10 Minutes	Not Yet All, A Little, Moderate, Very Much, Extreme
D	Practice Assessment Scale	Wipe The Face And Neck Properly Using A Hand Towel	Not Yet All, A Little, Moderate, Very Much, Extreme
D	Practice Assessment Scale	Make The Child Remain In The Fowler’s Position Or At Rest For 1-2 Hours After Inhalation	Not Yet All, A Little, Moderate, Very Much, Extreme
D	Practice Assessment Scale	Observe For The Present Signs And Symptoms After Steam Inhalation	Not Yet All, A Little, Moderate, Very Much, Extreme
